# Synthetic Melatoninergic Ligands: Achievements and Prospects

**DOI:** 10.1155/2014/843478

**Published:** 2014-02-23

**Authors:** N. V. Kostiuk, M. B. Belyakova, D. V. Leshchenko, V. V. Zhigulina, M. V. Miniaev

**Affiliations:** ^1^Department of Biology, Tver State Medical Academy, 4 Sovetskaya Street, Tver 170100, Russia; ^2^Department of Biochemistry, Tver State Medical Academy, 4 Sovetskaya Street, Tver 170100, Russia; ^3^Research Center, Tver State Medical Academy, 4 Sovetskaya Street, Tver 170100, Russia

## Abstract

Pineal hormone melatonin is widely used in the treatment of disorders of circadian rhythms. The presence of melatonin receptors in various animal tissues motivates the use of this hormone in some other diseases. For this reason, in recent years investigators continued the search for synthetic analogues of melatonin which are metabolically stable and selective to receptors. This review includes recent information about the most famous melatonin analogues, their structure, properties, and physiological features of the interaction with melatonin receptors.

## 1. Introduction

Almost since its opening in the mid-20th century epiphyseal hormone melatonin is seen as a valuable pharmacological agent. The results of the subsequent thorough and comprehensive study of the biochemical and physiological effects of melatonin have only confirmed this view. The positive results of the use of melatonin were obtained for the treatment of insomnia, circadian rhythm disorders associated with shift work, the change of time zones, and seasonal disorders [1–3]. The expediency of the use of melatonin is shown in the treatment of cardiovascular diseases, cancer, and other diseases [4, 5]. However, the widespread introduction of the drug in clinical practice has not been observed.

Experts believe that one of the limiting factors is the short half-life of melatonin. In recent years, two approaches to this problem are emerged. The first way is connected with the improvement of the pharmacokinetics due to the creation of medicinal forms of prolonged action. For example, the company Neurim Pharmaceuticals has produced drug called Circadin, mimicking physiological profile of epiphyseal hormone secretion. The second path involves the creation of more stable agonists, which also could selectively bind with a specific type of melatonin receptor. At present, this area is considered to be more promising. This topic is the focus of this review.

## 2. Melatonin

### 2.1. Structure and Biological Function of Melatonin

**Figure 1:**
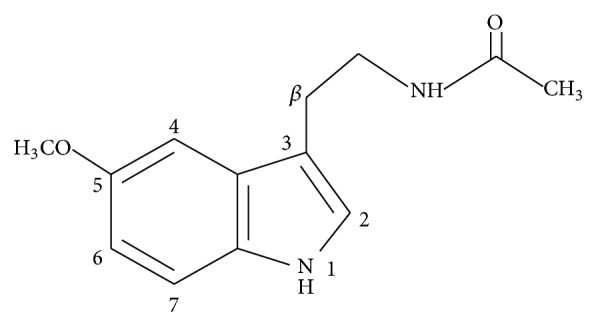
Structure of melatonin.

Melatonin (N-acetyl-5-methoxytryptamine) is a heterocyclic compound, derivative of indole (Figure 1).

In mammals, melatonin controls the set of physiological functions. It participates in the formation of circadian and seasonal rhythms [6–8], behavioral reactions [9, 10], and adaptation [11, 12]. It plays an important role in the regulation of reproductive function [13], the cardiovascular system [14, 15], immune reactions [16, 17], the restriction of the processes of cell proliferation, and tumor growth [18, 19]. Accumulated experimental data evidently demonstrate the influence of the pineal hormone on the state of protein, lipid, carbohydrate, and pigment metabolism [20–23]. The implementation of such diverse effects of melatonin is provided by the existence of numerous receptors and binding sites for the hormone.

### 2.2. Melatonin Biosynthesis

Melatonin is synthesized in the epiphysis from the essential amino acid tryptophan. First, by hydroxylation and decarboxylation serotonin is formed, which is then N-acetylated and O-methylated. The rate of melatonin synthesis is limited by the enzyme serotonin-N-acetyltransferase [1, 24]. The cascade of reactions is triggered in the darkness as a result of activation of *α*_1_- and *β*_1_-adrenoreceptors of pinealocytes by noradrenaline. This mechanism of regulation of the gland synthetic activity provides a circadian rhythm of melatonin secretion with a peak at night period [1, 25]. It was found that the effect of neurotransmitter is associated with increased transport of tryptophan in pinealocytes, formation of the terminal synthesizing enzyme hydroxy-O-methyltransferase, and activation of serotonin acetylation with simultaneous suppression of its oxidative deamination [26]. Synthesis of melatonin is affected by dopamine, glutamate, GABA, and serotonin. These compounds alter the activity of serotonin-N-acetyltransferase. Exogenous melatonin can also modulate the synthesis by both inhibiting and stimulating it [26, 27].

Epiphysis is not the only organ secreting melatonin. Cells producing this indole are found in the retina, Harderian gland, gastrointestinal tract, pancreas, respiratory tract, and thyroid and adrenal glands [26, 27]. Some scientists believe that extrapineal sources account for less than 20% of the body's melatonin, and others consider enterochromaffin cells as the main source of the hormone [26].

Newly synthesized melatonin is not accumulated in endocrine cells. It leaves the place of synthesis easily because of its ability to passively diffuse through the cell membrane. In blood, melatonin binds to proteins, preferably with a serum albumin and acidic glycoprotein *α*1 [28].

### 2.3. Degradation of Melatonin

As some compounds of indole nature, melatonin has a short half-life (30–50 minutes, depending on the species). In the liver, biotransformation is carried out by hydroxylation and subsequent formation of conjugates with sulfuric and glucuronic acids. In the other organs the hormone metabolism proceeds otherwise. The most common is deacetylation to form a 5-methoxytryptamine. In the retina, this compound is converted into 5-methoxyindoleacetic acid and 5-methoxytryptophol [29]. Mononuclear leukocytes are capable of regenerating N-acetyl serotonin and serotonin from melatonin [29]. While in brain cells the opening of melatonin pyrrole ring leads to formation of kynuramines [26]. Products of degradation are mostly excreted with the urine. Studies concerning the exchange of melatonin are reviewed fully [25, 28, 30].

## 3. Melatonin Receptors

In recent times a number of melatonin receptors have been identified. The greatest certainty is achieved for membrane (MT_1_ and MT_2_) and nuclear (RZR/ROR*α* and RZR/ROR*β*) receptors. Discussion regarding other melatonin binding sites is still far from complete. Binding sites were found in different parts of the cell: in the membranes (GPR50), in the cytosol (MT_3_), and in the mitochondria [31].

Receptors and binding sites of melatonin are distributed throughout the body. Their greatest number is noted in various brain structures, endocrine glands, and some peripheral organs [32, 33]. The distribution of receptors is organospecific. Several types of binding sites may occur in the cells of one organ. MT_1_ receptors are predominant [1]. Density and affinity of receptors undergo significant changes during the day. In the daytime, when the concentration of melatonin decreases, the number of its receptors is increasing [32, 33].

### 3.1. Membrane Receptors MT_1_ and MT_2_

In the cells of various mammalian species the two types of membrane receptors are revealed, MT_1_ and MT_2_, formerly known as Mel_1a_ and Mel_1b_. MT_1_ is found in the hypothalamus, the pituitary gland, cerebral cortex, thalamus, hippocampus, cerebellum, cornea and retina, arteries, heart, lungs, liver, kidney, adrenal gland, skin, and B and T lymphocytes [1, 33, 34]. MT_2_ receptors are localized in the hippocampus, the cerebellum, retina, arteries, heart, lungs, liver, small intestine, and skin [1, 32, 35].

In humans, the length of polypeptide chains is 350 and 362 amino acids, respectively. MT_1_ and MT_2_ molecules have high amino acid sequence homology (approximately 60%) [28]. In accordance with the characteristics of their spatial structure, the MT_1_/MT_2_ receptors belong to the family of rhodopsin/*β*_2_-adrenergic receptors [36]. They are based on seven transmembrane *α*-helices connected by a series of intra- and extracellular loops [34]. The extracellular N-terminal fragment has the glycosylation sites, and an intracellular C-terminal fragment includes phosphorylation sites [31, 37]. The palmitic acid residue can be attached to the cysteine residue of the fourth (intracellular) loop [38].

MT_1_ and MT_2_ receptors have high affinity to melatonin. For molecules isolated from human cells, *K*_*d*_ values are 80.7 and 383 pM, respectively [39]. Molecular mechanisms of melatonin binding to its receptors are far from sufficiently studied. It is assumed that the binding of 5-methoxyl group of melatonin may involve residues of histidine (His195 in the MT_1_ and His208 in the MT_2_) and valine (Val-192) of the fifth transmembrane helix. The structure of pocket for binding of melatonin's N-acetyl group appears to differ for two receptors. In the MT_1_ an important role belongs to the serine residues (Ser110 and Ser114) in the third transmembrane helix, whereas asparagine (Asn175) of the fourth helix has greater importance in the MT_2_ [28].

Recent works demonstrated polymorphisms of the MT_1_/MT_2_ receptors and related genes in human and animals. However, these mutations did not have clear phenotypic expression [28, 40].

Membrane receptors are associated with G-protein; however, depending on the tissue type intracellular signaling mechanisms may differ considerably. The most common is the suppression of cAMP synthesis by G_i_-proteins both sensitive and insensitive to pertussis toxin [34]. This reduces the activity of protein kinase A and the phosphorylation of several proteins. For example, it is shown that through receptors MT_1_ melatonin inhibits phosphorylation of transcription factor CREB (cAMP response element binding) [41].

The MT_1_ and MT_2_ receptors may be coupled with G_q/11_ protein, which does not use cAMP-dependent pathway but phosphoinositide signaling. The activation of phospholipase C (isoforms *β* and *γ*) in cells leads to increased amount of diglycerides, inositol trisphosphate and calcium ions, and protein kinase C activation [34]. Stimulation of protein kinase C causes multiple effects, including a cascade of mitogen-activated protein kinases (MAPKs). The phosphorylation was experimentally determined for the following enzymes related to the cascade: MEK1 and MEK2, JNK, and ERK1 and ERK2. With their activation the effect of melatonin on cell proliferation is linked [31, 42]. It is assumed that the activation of protein kinase C can be caused by the opening of calcium channels, dependent on G-protein, and the action of *βγ*-dimers formed by separating of *α*_i_-subunit from G-protein [31].

Another potentially important mechanism of melatonin signal transduction via the MT_2_ is the influence on the level of cGMP. While some researchers have observed an increase in the number of cGMP, probably due to inhibition of phosphodiesterase [43], others found reduced activity of soluble guanylate cyclase and cGMP production [44].

Through the MT_1_ and MT_2_ melatonin activates potassium channels GIRK (G protein-coupled inwardly rectifying potassium channels) [45] and BK_Ca_ (large-conductance Ca^2+^-activated K^+^ channels). The opening of potassium channels may underlie the vasomotor and neurotrophic effects of epiphyseal hormone [31].

Molecules of MT_1_ and MT_2_ as many receptors coupled to G-proteins are capable of dimerization. MT_1_ homodimers and MT_1_/MT_2_ heterodimers are formed in several times lighter than homodimers MT_2_ [46]. Therefore, it seems quite possible to form heterodimers in the retina, hippocampus, and neurons of the suprachiasmatic nuclei in hypothalamus, where both types of melatonin receptors are expressed. It is suggested that the formation of heterodimers may be important for the realization of the signal via the MT_2_ receptors [32].

Recent studies have shown that MT_1_ and MT_2_ form complexes with certain intracellular proteins. Some of them are associated with both receptors (filamin and IRS4, insulin receptor substrate 4), while others show greater selectivity. MT_1_ receptor specifically interacts with phosphodiesterase, protein elongation factor EEF-1B*γ*, Rac1 (Ras-related C3 botulinum toxin substrate 1), and Rap-1A (Ras-related protein 1A). MT_2_ receptor binds to protein phosphatase 2C*γ* (PP2C*γ*) and catenin *δ*1. The biological role of this phenomenon is still unclear [32].

It is believed that modification of the affinity and number of melatonin receptors is an important component of the mechanism which regulates the circadian rhythm. Prolonged exposure to hormone leads to desensitization of membrane receptors [47], probably as a result of phosphorylation of the C-terminus [38]. Internalization of receptors occurs in the usual manner. The binding of MT_1_ and MT_2_ with the protein *β*-arrestin [48] facilitates interaction with clathrin adaptors, which further leads to isolation of clathrin-coated vesicle containing “extra” receptors [49]. After treatment with physiological concentrations of hormone, a long time (about 8 hours) is required to restore the number of membrane receptors. This process is largely dependent on the synthesis of new protein molecules [50].

### 3.2. Nuclear Receptors

Nuclear receptors for melatonin RZR/ROR*α* and RZR/ROR*β* are also referred as NR1F1 and NR1F2 according to the unified nomenclature. They belong to a family of the retinoid receptors and have lower affinity for melatonin than MT_1_ and MT_2_ [31, 51]. RZR/ROR*α* is localized mainly in the cerebellum, thalamus, hippocampus, lymphocytes, and skin, while the RZR/ROR*β* is found in the retina, pineal gland, pituitary gland, hypothalamus, thalamus, and spinal cord [51].

The nuclear receptors have a typical domain organization. C-terminal domain provides the ligand attaching. It is also responsible for receptor dimerization. However, unlike other members of the family of nuclear receptors, RZR/ROR*α* and RZR/ROR*β* can function as monomers. DNA-binding domain, composed of two “zinc fingers,” recognizes hexanucleotide RGGTCA (wherein R = A or G), adjacent to the 5′-end to the AT rich sequences [52].

It is assumed that the nuclear melatonin receptors are responsible for the manifestation of the hormone immunomodulatory action as it enhances the syntheses of interleukins and *γ*-interferon by T lymphocytes [53]. In B lymphocytes melatonin inhibits the formation of 5-lipoxygenase, a key enzyme in the synthesis of leukotrienes which are involved in allergic and inflammatory response [54]. Antiproliferative effects of melatonin are also related with the activation of nuclear receptors, because specific hexanucleotides, recognized by RZR/ROR, were detected in the promoters of some cell cycle regulatory proteins, for example, p21WAF1/CIP1 [51].

### 3.3. Melatonin Binding Sites

#### 3.3.1. The Melatonin Binding Site GPR50

The polypeptide chain of this protein is 618 amino acid residues in length. Binding site GPR50 has a high (about 45%) amino acid sequence homology with MT_1_ and MT_2_ and structural features specific for the melatonin receptors [55]. GPR50 was detected in the hypothalamus, the pituitary gland, hippocampus, retina, testes, and kidneys [32]. Despite the fact that GPR50 was cloned in 1996, its functions are still poorly understood. Recent studies have shown that GPR50 readily forms heterodimers with receptors MT_1_ and MT_2_. As part of such heterodimers, the MT_2_ retains its properties, while the affinity of MT_1_ to agonists dramatically reduced [32]. Association of MT_1_ with GPR50 also prevents interaction with G-protein and a *β*-arrestin that ultimately affects the intracellular signal transduction and internalisation of receptors [56].

#### 3.3.2. Melatonin Binding Site MT_3_

Discovery of this binding site, found in the liver, kidney, and brain [57], has generated a large number of still unsolved issues. MT_3_ initially was treated as a membrane receptor, but later it turned out that about 90% of these molecules are localized in the cytoplasm [58]. MT_3_ demonstrates low affinity to hormone. *K*_*i*_ value is measured in nanomoles that exceeds the concentration of melatonin in the blood. With increasing temperature the affinity decreases, and to record the formation of complex MT_3_-2-(^125^I)-melatonin is practically impossible at 37°C [57, 58]. Despite the efforts, the mechanisms of signal transduction from the MT_3_ have not been revealed yet. These findings raise the validity of the MT_3_ receptor as classical receptors [59].

As a result of numerous studies MT_3_ was identified as quinone reductase QR2 (EC 1.10.99.2). The biological role of the enzyme is unknown but it is assumed that it is involved in neutralization of toxic quinones. On this basis it has been hypothesized that antioxidant properties of melatonin are related with the activity of QR2 [31]. Method of X-ray analysis shows that melatonin attaches the enzyme not in allosteric but in the active center [60]. In this case, some researchers consider a role of melatonin as a substrate, electron donor in neutralization of active oxygen radicals [59], and the others believe that it acts as competitive inhibitor of the enzyme [60]. The data obtained with the help of nuclear magnetic resonance confirm the second opinion [61].

## 4. Synthetic Ligands of Melatonin Receptors

### 4.1. Search Strategy of New Ligands

In the past two decades, a large number of ligands for melatonin receptors have been synthesized. Structure-activity relationships (SARs) of melatonin derivatives have been comprehensively analyzed [62–65], and we only summarize general strategies used in the development of melatoninergic ligands.

Large-scale search for effective melatonin receptor agonists was started in the late 80s. The work was done on tissue samples, which contain, as it turned out later, a heterogeneous set of melatonin binding sites, so the dependence of the “structure-activity” was considered without taking into account the differences in the structure of the receptor [65]. The impetus for further research was the cloning of MT_1_ and MT_2_ and the selection of cell lines expressing only a certain type of melatonin receptor (lines CHO, COS-7, HEK293, and NIH3T3) [62]. Retesting of known ligands on cell lines in most cases showed their low selectivity for MT_1_ and MT_2_. In addition to traditional experimental methods, the computer simulation of ligand is used more increasingly, while the existing algorithms do not allow distinguishing MT_1_ and MT_2_ [65]. The formulas of some specific ligands of melatonin receptors are given in Table 1.

First agonists were obtained by modification of melatonin structure. These agonists were used to specify positions important for interaction with the receptor. Position 6 is determinative for binding as well as methoxy group (position 5), and amide group of side chain. Introduction into the molecule of large substituents in the positions 1 and *β* leads to dramatic decrease in affinity for the receptor. In modifications of position 2 the biological activity is maintained. Potent agonists are molecules containing methyl, phenyl, or halides in this position. Radioactive form of 2-iodomelatonin (Table 1, structure 1) has become the standard for the study of melatonin receptors [65]. Bioisosteres were obtained by replacement of nitrogen atom in the pyrrole ring by oxygen and sulfur [6]. Shift of nitrogen from 1 to 3 positions appears admissible [73].

Positive results are shown for molecules with partial restriction of conformational mobility [62]. In such compounds, indole rings are included in a system of three or four conjugated cycles (structures 4 and 5). It was shown that the indole nucleus may be substituted by other aromatic moieties. One of the most successful substitutions was a replacement of melatonin heterocycle by naphthalene (structures 8 and 9) [74]. It was found that the presence of the ligand molecule of condensed rings is not strictly necessary. Good affinity appears in substances in which cycles are separated by short linear bridges [75]. An interesting approach was used to create “dimeric” ligands, a symmetric structures obtained by association of two molecules of known agonists (structure 10 and 11) [70, 76]. Effective agonists occur among derivatives of indole, benzofuran, naphthalene, and tetralin. Work in creation of novel agonists is still going on [77–82].

Search of selective agonists is essential for determination of the role of each receptor type in the implementation of the biological effects of melatonin. Although significant structural similarity in MT_1_ and MT_2_, in recent years ligands were collected which specifically bind to one of these receptors. For such a highly selective ligands, the *K*_*i*_ values are usually different in the tens or hundreds of times (Table 1). There are cases where the influence of the ligand on the functional activity of membrane receptors is opposite. For example, compound 5-HEAT (structure 2) is an agonist of MT_1_ and acts as an antagonist of MT_2_ [66]. The pharmacological profile of the MT_3_ is very different from the MT_1_ and MT_2_. The 5-MCA-NAT (structure 3), prazosin, and N-acetyltryptamine exhibit the highest selectivity [67]. Specific ligand for the nuclear receptor is a thiazolidinedione CGP52608 (structure 12) and structurally related molecules [83].

### 4.2. Prospects for Clinical Use of the Melatonin Receptors Agonists

Currently, only membrane melatonin receptor agonists have clinical interest. Although the number of synthesized and tested ligands of MT_1_/MT_2_ amounts to hundreds, only a few compounds have reached the stage of clinical trials. Ramelteon (Rozerem) was developed by the pharmaceutical company Takeda and approved in the US in 2005. Agomelatine (Valdoxan, Melitor, Thymanax) was developed by the pharmaceutical company Servier and approved in Europe in 2009. Two melatonin agonists, Tasimelteon and TIK-301, have received orphan drug designation and are in clinical trials in the United States. Tasimelteon was developed by Vanda Pharmaceuticals, and phase III of its clinical trial was completed in 2010. TIK-301 was designed originally by Eli Lilly and Company, and since 2007 the trials have been undergone in Tikvah Pharmaceuticals. In February 2013, Neurim Pharmaceuticals reported positive results of phase II of trials for piromelatine (Neu-P11).

Ramelteon, N-{2-[(8S)-1,6,7,8-tetrahydro-2H-indeno[5,4-b]furan-8-yl]ethyl} propanamide, is the first among the melatonin receptor agonists approved by FDA. The affinity of ramelteon for MT_1_ and MT_2_ exceeds the affinity of the natural ligand (Table 1), and so in cell cultures ramelteon demonstrates better result in the inhibition of forskolin-induced synthesis of cAMP. Significance of IC50 (half maximal inhibitory concentration) of ramelteon and melatonin varies in 3–18 times depending on the type of receptor [39].

When orally administered, ramelteon is rapidly absorbed. Peak of drug concentration in plasma is achieved in approximately 1 h after administration [84]. The drug is metabolized in the liver by hydroxylation and subsequent conjugation with glucuronic acid [85]. To date, 4 metabolites are identified. It was found that the major metabolite (M-II) hydroxylated at position C2 of propionyl residue is weak agonist of MT_1_/MT_2_ receptor. Its elimination half-life is from 2 to 5 hours. According to some estimates, which take into account the duration of the metabolite existence, half-life of ramelteon may reach 2.6 hours [86]. The existence of a long-lived active metabolite should contribute to the manifestation of the pharmacological effects of ramelteon that should be considered as a decisive advantage for the use of the drug in clinical practice.

During the preclinical and clinical trials it was shown that ramelteon promotes sleep without causing any significant side effects. The drug does not affect the coordination of movements, memory, and learning ability. It has no sedative effect, so sleep induced by the drug is indistinguishable from natural sleep [87]. It appears that the absence of undesirable side effects of ramelteon is explained by inability to bind receptors of benzodiazepine, dopamine, serotonin, and opioids [88]. Ramelteon reduces sleep latency and increases total sleep time without causing hangover, addiction, and withdrawal effects [87].

Due to proven clinical effectiveness and safety, ramelteon is considered as the fourth-generation drug for the treatment of primary insomnia and insomnia associated with circadian rhythms [89]. Other possible fields of ramelteon application require a detailed study. More detailed information on the results of preclinical and clinical testing can be found in the reviews [87, 89, 90].

Agomelatine, N-[2-(7-Methoxy-1-naphthyl)ethyl], is a derivative of naphthalene. The drug has a high affinity to MT_1_/MT_2_ receptors comparable with melatonin (Table 1).

When administered per os, agomelatine, as well as ramelteon, has low bioavailability. Peak of drug concentration in blood plasma is observed within 1-2 hours. Almost all of the molecules of agomelatine are associated with blood proteins. Biotransformation of agomelatine occurs mainly in the liver to form hydroxylated and demethylated derivatives. Four metabolites of agomelatine are identified: 3-hydroxy-, 7-methoxy-, 7-desmethyl-, and dihydrodiol-agomelatine [91]. These compounds are not biologically active and are excreted in the urine. Half-life is less than 2 hours [89].

Binding to MT_1_/MT_2_ receptors, agomelatine synchronizes circadian rhythms in animals with delayed sleep phase syndrome [86]. In clinical trials, agomelatine also demonstrates positive phase shifting properties; it induces a phase advance of sleep, body temperature decline, and melatonin onset [86]. Agomelatine is a selective antagonist of the serotonin receptors 5-HT_2C_ [92]. Because of this peculiarity, the drug can be used for the treatment of depressive disorders [93–95]. Therefore, agomelatine is a unique tool against insomnia caused by depression. A detailed review of the pharmacological properties of the drug can be found in the literature [89, 95, 96].

Tasimelteon (VEC-162, BMS-214778) is a derivative of propanamide, N-[[(1R, 2R)-2-(2,3-dihydro-1-benzofuran-4-yl)cyclopropyl]methyl] propanamide. The drug demonstrates a higher affinity for melatonin receptors than natural ligand (Table 1). Unlike melatonin, ramelteon, and agomelatine, binding of MT_2_ is easier than MT_1_ [30].

In plasma, tasimelteon circulates predominantly in protein-bound form (<91%). The drug is distributed throughout the body. Its metabolism occurs in the liver by hydroxylation and dehydrogenation [97].

In clinical trials, tasimelteon improved sleep latency, sleep efficiency, and wake after sleep onset (i.e., sleep maintenance). The drug exhibits a good safety profile with no significant side effects in comparison with placebo [98]. On May 31, 2013, Vanda Pharmaceuticals Inc. presented tasimelteon as a new drug for the treatment of Non-24-hour sleep-wake disorder.

Neu-P11 (piromelatine, N-(2-(5-methoxy-1H-indol-3-yl)ethyl)-4-oxo-4H-pyran-2-carboxamide) is a new potential drug for the treatment of insomnia [99]. Neu-P11 is also agonist of melatonin receptors MT_1_/MT_2_ and serotonin receptors 5-HT_1A/1D_ and so has the properties which are weakly expressed in other melatoninergic drugs. Neu-P11 has antidepressant and anxiolytic properties similar to those of melatonin [100]. It appears that preparation will find application in the treatment of affective disorders. Neu-P11 is able to attenuate cognitive impairment that may be promising in the treatment of Alzheimer's disease [101]. In addition, positive effects of Neu-P11 on glucose level and triglycerides by increasing the sensitivity of cells to insulin are shown [102].

## 5. Conclusion

After half a century of studying melatonin, it is considered as an integral part of the homeostatic mechanisms of the organism and a hormone involved in regulating a large number of various physiological processes. Definition of the melatonin receptors structure, the discovery of signaling mechanisms, the establishment of cell lines and animal models, and synthesizing only MT_1_ or MT_2_, all this contributed to the understanding of the role of melatonin and its receptors in the modulation of visual, circadian, endocrine, and immune functions.

The accumulated information served as a catalyst for the creation of synthetic melatonergic ligands. Over the past three decades there were synthesized and tested hundreds of molecules, which specifically bind to melatonin receptors. Functional groups of melatonin, which are critical for binding to the receptors, have been clarified that allowed a realization of the systematic approach to the synthesis of new ligands. Radiolabeled ligands, selective agonists and antagonists to MT_1_ and MT_2_ receptors, were already used as a tool for studying of melatonin functioning. Some melatonin agonists have obvious pharmacological value. Currently five compounds (ramelteon, agomelatine, tasimelteon, Neu-P11, and TIK-301) have reached a stage of clinical trials, two of them (ramelteon and agomelatine) gained approval for clinical use as drugs for the treatment of insomnia and violations of circadian rhythms.

However, the development of the new melatoninergic ligands is far from complete. It is necessary to expand the spectrum of high-selective agonists and antagonists, which could be used both for scientific research and in medical practice. It seems to be promising a study of synergistic relationships between melatonin receptors and receptor of neurotransmitters, such as serotonin. This dual activity detected in agomelatine made it effective in treatment of insomnia caused by depression. Another important direction is the search of melatonergic ligands with other pharmacological activities. Based on the biological role of melatonin, among its ligands we can expect the existence of potential drugs for treatment of oncological diseases and metabolic and endocrine disorders.

## Figures and Tables

**Table 1:** Synthetic ligands at melatonin receptors.

Number	Compound∗	Type of ligand	Reference
Melatonin derivatives			
**1**	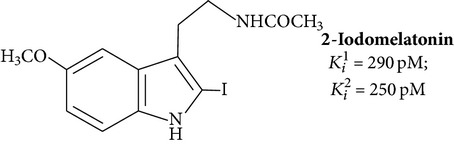	Agonist MT_1_ and MT_2_	[65]
**2**	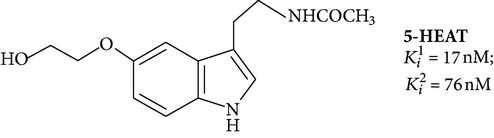	Agonist MT_1_, antagonist MT_2_	[66]
**3**	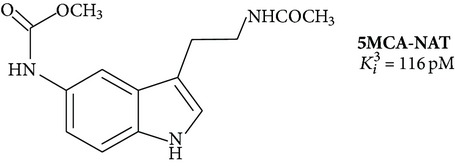	Agonist MT_3_	[67]

Tri- and tetracyclic compounds			
**4**	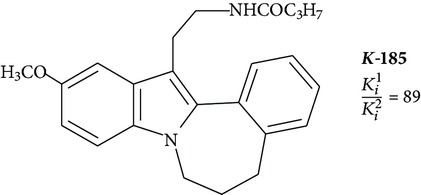	Antagonist MT_2_	[68]
**5**	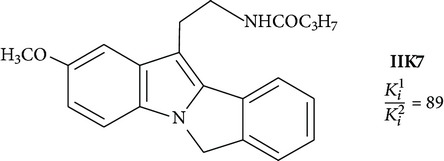	Agonist MT_2_	[68]
**6**	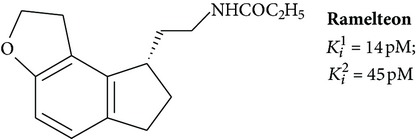	Agonist MT_1_ and MT_2_	[65]
**7**	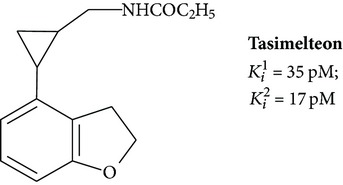	Agonist MT_1_ and MT_2_	[30]

Naphthalene and tetralin analogues			
**8**	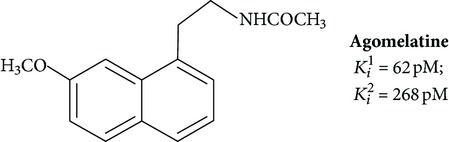	Agonist MT_1_ and MT_2_	[69]
**9**	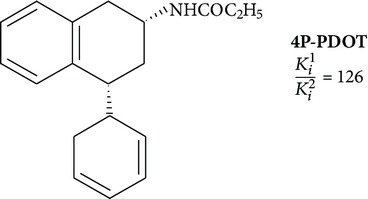	Antagonist MT_2_	[70]

“Dimeric ligands”			
**10**	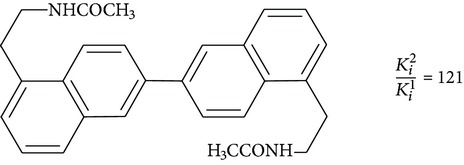	Agonist MT_1_	[70]
**11**	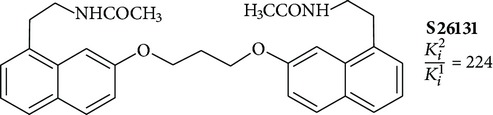	Antagonist MT_1_	[71]

Thiazolidine analogues			
**12**	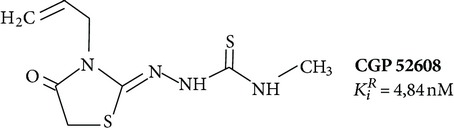	Agonist RZR/ROR	[72]

^*^For ligands with low selectivity to the MT_1_ and MT_2_ and also for the MT_3_ and RZR/ROR ligands, the values of the dissociation constants for receptor complex are shown as *K*_*i*_^1^, *K*_*i*_^2^, *K*_*i*_^3^, *K*_*i*_^*R*^, respectively. For ligands of high selectivity, the ratios of the dissociation constants are given, *K*_*i*_^1^/*K*_*i*_^2^ or *K*_*i*_^2^/*K*_*i*_^1^.
